# Genomic prediction of male fertility in Brown Swiss cattle

**DOI:** 10.3168/jdsc.2023-0533

**Published:** 2024-04-20

**Authors:** Hendyel A. Pacheco, Attilio Rossoni, Alessio Cecchinato, Francisco Peñagaricano

**Affiliations:** 1 Department of Animal and Dairy Sciences, University of Wisconsin–Madison, Madison, WI 53706; 2 Italian Brown Breeders Association, Bussolengo, Verona 37012, Italy; 3 Department of Agronomy, Food, Natural Resources, Animals and Environment, University of Padova, Legnaro, Padua 35020, Italy

## Abstract

Bull fertility has been recognized as an important factor affecting dairy herd fertility. The objective of this study was to assess the feasibility of predicting male fertility in Brown Swiss cattle using genomic data. The dataset consisted of 1,102 Italian Brown Swiss bulls with sire conception rate (SCR) records and genotype data for roughly 480k SNP. The analyses included the use of linear kernel-based regression models fitting all SNPs or incorporating markers with large effect. Predictive performance was evaluated in 5-fold cross-validation using the correlation between observed and predicted SCR values and mean squared error of prediction. The entire SNP set exhibited predictive correlations around 0.19. Interestingly, the inclusion of 2 markers with large effect yielded predictive correlations around 0.32. Overall, using linear kernel-based models fitting markers with large effect is a promising approach. Our findings could help Brown Swiss breeders make enhanced genome-guided management and selection decisions on male fertility.

## Highlights

• The entire SNP set exhibited predictive correlations around 0.19.
• The inclusion of 2 markers with large effect yielded predictive correlations around 0.32.
• Genomic prediction of dairy bull fertility is possible and deserves further attention.

## Graphical Abstract

**Figure:**
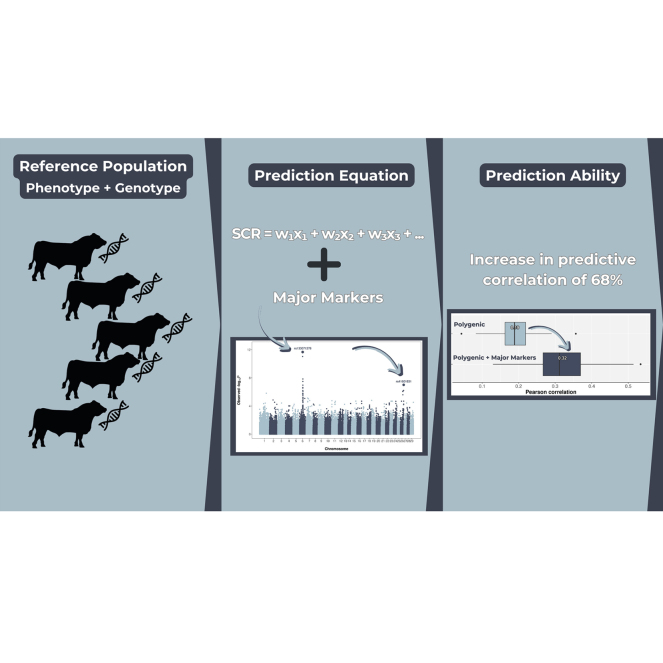
**Summary:** The service sire has been recognized as an important factor affecting reproductive efficiency in dairy cattle. Here, we assessed the feasibility of predicting bull fertility in Italian Brown Swiss cattle using genomic data. The analysis included the use of alternative models fitting all single nucleotide polymorphisms (SNPs) or incorporating markers with large effect. The entire set of SNPs yielded predictive correlations of 0.19, while the inclusion of 2 markers with large effect increased predictive correlations to 0.32. Early genomic predictions of bull fertility could help the dairy industry make enhanced genome-guided selection and management decisions, such as early culling of predicted subfertile bull calves.

Genome-wide dense markers, such as SNPs, have facilitated so-called genome-enabled prediction, which aims to predict unobserved genetic values or yet-to-be-observed phenotypes using genomic information (Meuwissen et al., 2001). Genomic prediction has revolutionized dairy cattle breeding worldwide because it allows breeders to make accurate selection decisions at an early age, even when neither the animal nor its offspring has been assessed for the phenotypes of interest. Indeed, by shortening the generation interval and increasing the accuracy and intensity of selection, genomic prediction in dairy cattle has at least doubled annual genetic gains for economically important traits (García-Ruiz et al., 2016).

Various methods have been proposed to estimate genetic effects and predict complex traits using genomic data (Meuwissen et al., 2001; Gianola et al., 2006; VanRaden, 2008; Misztal et al., 2009). Different strategies have been suggested to improve the accuracy of genomic predictions, such as selecting markers with large effect (Weigel et al., 2009; Nani et al., 2019). Indeed, the identification of variants and genes that affect the phenotype of interest and the incorporation of this information into prediction models can improve the accuracy of genomic predictions. Note that genomic prediction is traditionally viewed as a black-box tool, but it is now recognized that the use of prior biological information can enhance predictive performance and model robustness (Koltes and Peñagaricano, 2019).

The advent of genomics has the potential to improve bull fertility in dairy cattle. In fact, we have shown that the use of biologically informed models can improve the accuracy of genomic predictions for service sire fertility. These approaches have been successfully applied in both Holstein and Jersey cattle (Abdollahi-Arpanahi et al., 2017; Nani et al., 2019; Rezende et al., 2019, 2020; Pacheco et al., 2020). Of special interest, there is a substantial variation in fertility among Brown Swiss bulls (Pacheco et al., 2021) and recent studies have identified genomic regions and individual genes that have a major effect on Brown Swiss bull fertility (Hiltpold et al., 2020, 2021; Mapel et al., 2022; Pacheco et al., 2022, 2023). The incorporation of this information into prediction models could lead to more accurate and robust genomic predictions of Brown Swiss male fertility. As such, our first objective was to assess the prediction of bull fertility in the Italian Brown Swiss cattle population using 480k SNP markers spanning the entire genome. Our second objective was to evaluate the potential benefits of incorporating markers with large effect into the genomic prediction models.

Phenotypic data consisted of sire conception rate (**SCR**) records from a total of 1,102 Italian Brown Swiss bulls. Sire conception rate records were calculated using cow field data, considering factors related to the bull under evaluation and factors (nuisance variables) associated with the cow that receives the unit of semen (Kuhn and Hutchison, 2008; Kuhn et al., 2008; Pacheco et al., 2021). Estimates of SCR were calculated as the deviation from the mean, which is set as 0, so each 1-point difference reflects 1% more conception rate. For bulls with multiple SCR records, the most reliable SCR record (i.e., the SCR record with the most breedings) was used in this study. Sire conception rate values in the Italian Brown Swiss population ranged from −22.3% to 9.9%, with the number of breedings ranging from 50 to 8,110.

Genotype data for 572,527 SNP markers located in autosomal chromosomes were available for all the 1,102 Italian Brown Swiss bulls with SCR records. This is a subset of SNP markers available in the BovineHD Genotyping BeadChip (Illumina Inc.) with good imputation properties, and thus high imputation accuracy. The SNP information, including chromosome and position, was based on the bovine reference genome ARS-UCD-1.2. The SNP markers that were monomorphic, had minor allelic frequency below 1%, and had call rate less than 99% were removed from the dataset. All bulls had a call rate above 95%. After quality control, a total of 481,839 SNP markers were retained for subsequent genomic analysis.

We evaluated the feasibility of predicting bull fertility in the Brown Swiss population. To assess the predictive power of the entire high-density SNP dataset, a whole-genome prediction model was employed on the Italian Brown Swiss data, consisting of 1,102 animals and 481,839 SNP markers. Our previous research showed that 2 of the 481,839 SNPs (*rs133071278* and *rs41601831*) have major effects in bull fertility in Brown Swiss cattle (Figure 1; Pacheco et al., 2022). Here, these 2 SNPs were coded as 0 or 1 to represent the effect of having 2 copies of the recessive allele, and were fitted as fixed effects in an alternative whole-genome prediction model. Note these 2 markers are not the actual causal variants but are in linkage disequilibrium with the causal variants.

**Figure 1:**
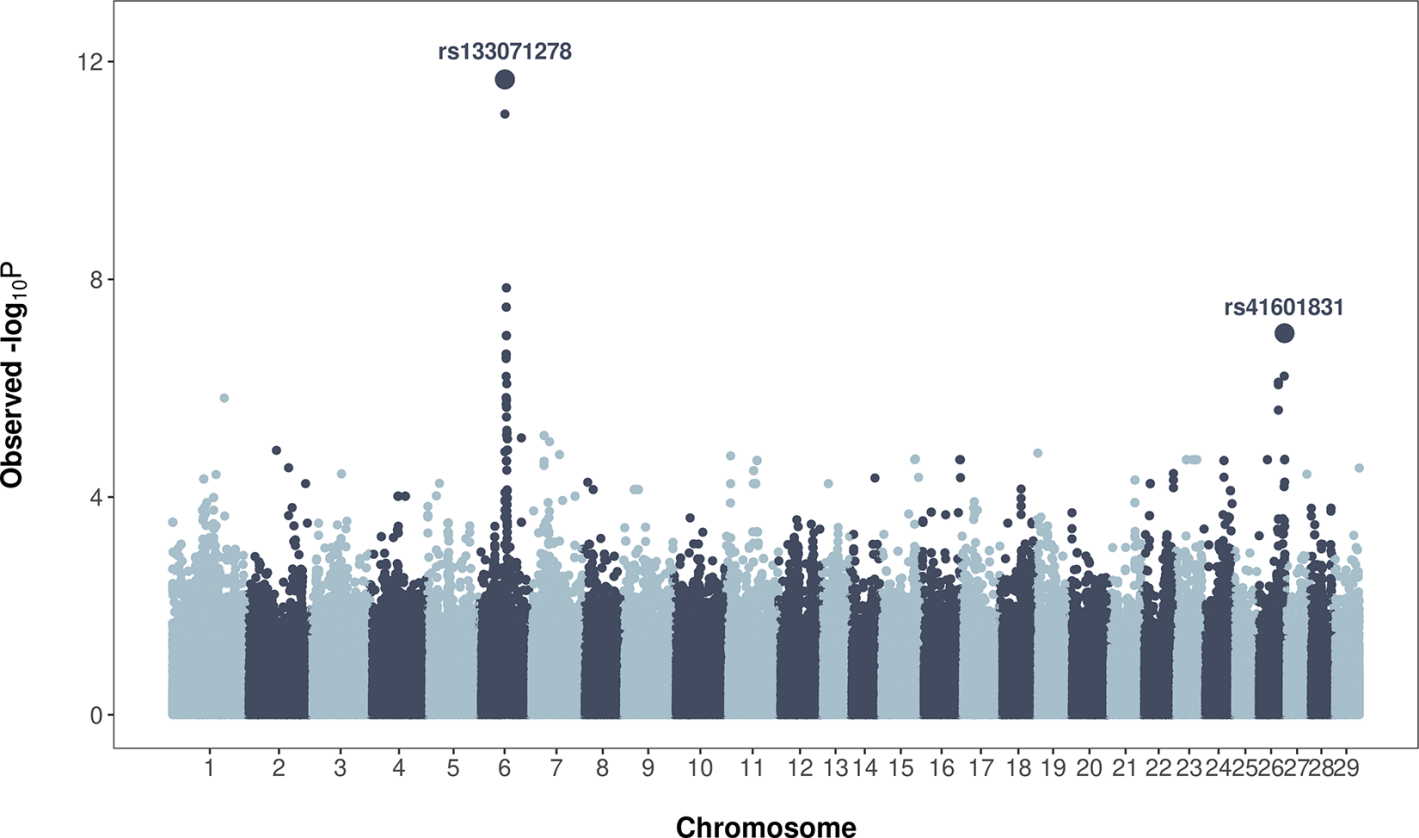
Whole-genome scan for recessive effects on sire conception rate. Manhattan plot showing the SNP significance across the bovine genome for recessive effects on sire conception rate in the Italian Brown Swiss bull population.

The predictive ability of the different approaches was evaluated using Bayesian reproducing kernel Hilbert spaces (**RKHS**) regression models (Gianola and van Kaam, 2008; Morota and Gianola, 2014). The analyses were implemented under the general kernel-based regression model:

**y** = **Xb** + **Kα** + **e**,

where **y** is the vector of phenotypic records (SCR values), **b** is the vector of fixed effects including a general intercept (µ), **X** is the design matrix relating fixed effects to SCR records, **K** is an *n* × *n* kernel matrix indexed by the SNP genotype matrix, **α** is the vector of RKHS regression coefficients estimated as the solution that minimizes l(α|λ)=(y−Kα)′(y−Kα)+λα′Kα, where *λ* is the regularization parameter (*λ* = σe2/σe2σg2σg2), and **e** is the error term. The random effects **α** and **e** were distributed as $\alpha \&sim;\;N\left(0,K^{-1}\sigma_{g}^{2}\right)$ and $e\&sim;\;N\left(0,R^{-1}\sigma_{e}^{2}\right),$ where $\sigma_{g}^{2}$ and $\sigma_{e}^{2}$ are the genetic and residual variances, respectively, and **R** is a diagonal matrix with its elements representing reliabilities of the SCR records.

Kernel-based genomic prediction models were fitted using linear reproducing kernels (**K**). The linear (**K**_L_) kernel, which is equivalent to the well-known additive genomic relationship matrix formulated by VanRaden (2008), takes the form **K**_L_ = **SS**′/*p*, where **S** is a matrix of centered and standardized SNP genotypes and *p* represents the number of SNP. The models were implemented in a Bayesian framework via Markov chain Monte Carlo. All the analyses were performed using the R package Bayesian Generalized Linear Regression (Pérez and de Los Campos, 2014; Pérez-Rodriguez and de Los Campos, 2022). The predictive ability of the different kernel models was assessed by 5-fold cross-validation with 10 repetitions. The ability to predict yet-to-be observed SCR values was assessed using the Pearson product-moment correlation coefficient and the mean squared error of prediction (**MSEP**).

Figure 2 shows the predictive ability of linear kernel-based regression models fitting the whole-genome model, “Polygenic,” and the “Polygenic + Major Markers” model that includes 2 significant recessive SNPs fitted as fixed effects. The “Polygenic” model exhibited an average correlation between observed and predicted SCR values of 0.19, and a MSEP equal to 22.11. The “Polygenic + Major Markers” model delivered an average predictive correlation equal to 0.32 and MSEP equal to 20.34, representing an increase in predictive performance of about 68%. These SNPs, *rs133071278* and *rs41601831*, show allele frequencies of 0.29 and 0.31, respectively, in the Italian Brown Swiss population. Pacheco et al. (2022) reported that these significant nonadditive markers are near genes directly involved in male fertility, including sperm motility, acrosome reaction, and embryonic development. Note that incorporating prior information into genomic models from either public QTL databases (Zhang et al., 2014) or the current dataset (Zhang et al., 2015) has been shown to improve the accuracy of genomic predictions. This aligns with the findings of Lopes et al. (2017), who demonstrated that including markers with relatively large effects enhanced model predictive ability for the number of teats in different pig populations. This improvement in predictive accuracy was also observed when large effect QTL was added as a fixed effect in the predictive models in plants (Chen et al., 2023). These results emphasize the benefit of incorporating previous biological knowledge into predictive models, which leads to more accurate predictions.

**Figure 2:**
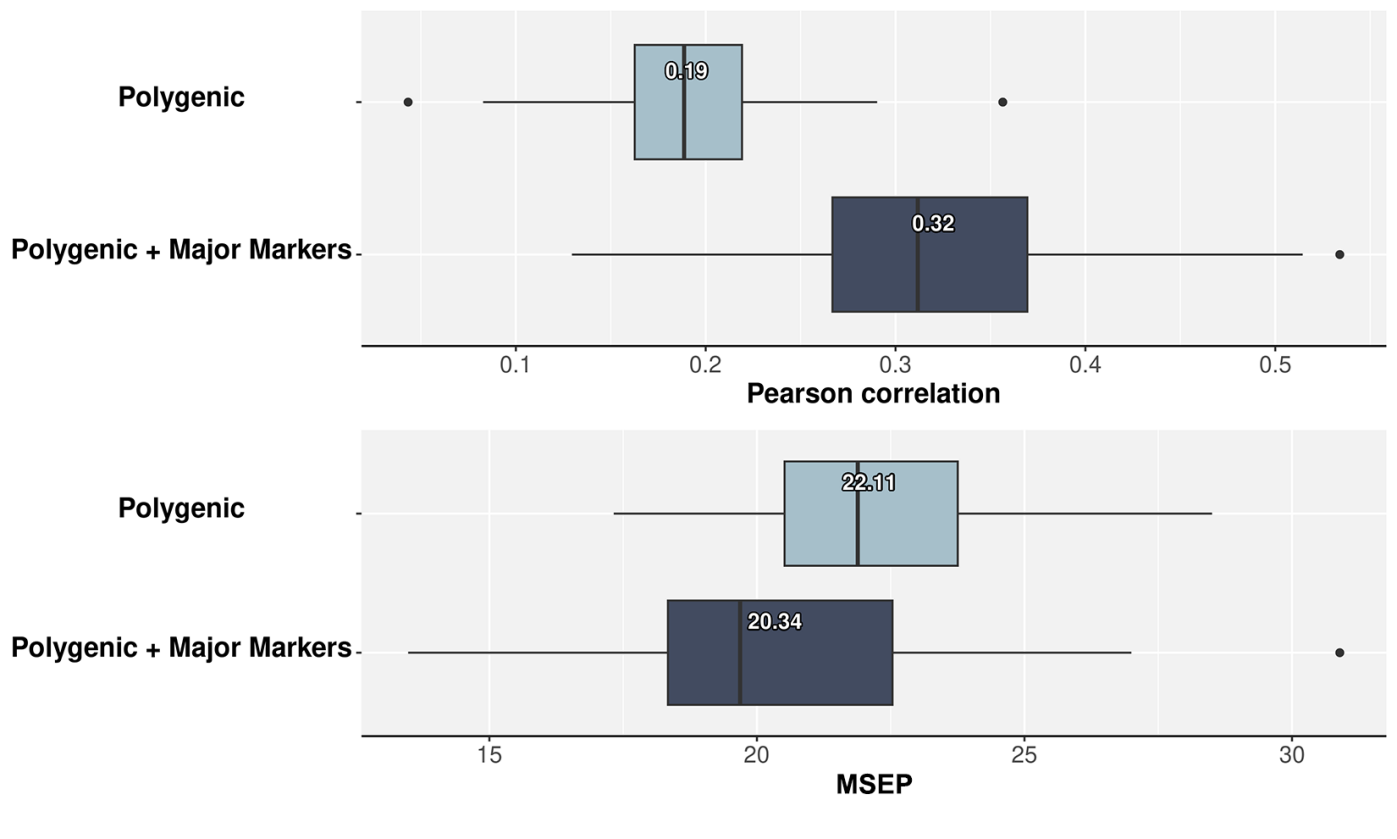
Genomic predictions within the Italian Brown Swiss population using alternative whole-genome predictive models. Predictive correlation (top) and mean squared error of prediction (bottom) were calculated using 5-fold cross-validation with 10 replicates. Light blue boxes represent the “Polygenic” model that includes the whole SNP dataset (481,839 SNPs). Dark blue boxes represent the “Polygenic + Major Markers” model that includes 2 major SNP markers fitted as fixed effects. The left and right of the box represent first and third quartiles; the vertical line denotes the median; the value denotes the mean; the whiskers correspond to 1.5 × interquartile distance; and dark dots are outliers.

Our study reinforces the idea that genomic prediction of male fertility is feasible in dairy cattle (Peñagaricano, 2024). Note that SCR records are available only after the bulls are in the market, and hence, early genomic predictions can help Brown Swiss breeders make enhanced selection decisions, such as early culling of predicted subfertile bull calves. Our study also showed that the inclusion of markers with large nonadditive effects can markedly improve the prediction of dairy sire fertility. Overall, this study provides new strategies for selecting and managing service sire fertility in Brown Swiss cattle.
